# A Novel Pathogenic Variant in *CARMIL2* (*RLTPR*) Causing CARMIL2 Deficiency and EBV-Associated Smooth Muscle Tumors

**DOI:** 10.3389/fimmu.2020.00884

**Published:** 2020-06-18

**Authors:** Jennifer R. Yonkof, Ajay Gupta, Cesar M. Rueda, Shamlal Mangray, Benjamin T. Prince, Hemalatha G. Rangarajan, Mohammad Alshahrani, Elizabeth Varga, Timothy P. Cripe, Roshini S. Abraham

**Affiliations:** ^1^Division of Allergy and Immunology, Department of Pediatrics, Nationwide Children's Hospital, Columbus, OH, United States; ^2^Division of Hematology, Oncology and Blood and Marrow Transplant, Nationwide Children's Hospital, Columbus, OH, United States; ^3^Department of Pathology and Laboratory Medicine, Nationwide Children's Hospital, Columbus, OH, United States; ^4^Division of Hematology and Oncology, Department of Pediatrics, Nationwide Children's Hospital, Columbus, OH, United States; ^5^Department of Pediatric Hematology-Oncology, Riyadh Military Hospital, Riyadh, Saudi Arabia

**Keywords:** primary immunodeficiencies, immune dysregulation, CARMIL2 deficiency, RLTPR, DOCK8 deficiency, EBV-associated smooth muscle tumor, Early-onset inflammatory bowel disease (IBD)

## Abstract

CARMIL2 deficiency is a rare combined immunodeficiency (CID) characterized by defective CD28-mediated T cell co-stimulation, altered cytoskeletal dynamics, and susceptibility to Epstein Barr Virus smooth muscle tumors (EBV-SMTs). Case reports associated with EBV-SMTs are limited. We describe herein a novel homozygous *CARMIL2* variant (c.1364_1393del) in two Saudi Arabian male siblings born to consanguineous parents who developed EBV-SMTs. CARMIL2 protein expression was significantly reduced in CD4+ T cells and CD8+ T cells. T cell proliferation on stimulation with soluble (s) anti-CD3 or (s) anti-CD3 plus anti-CD28 antibodies was close to absent in the proband, confirming altered CD28-mediated co-signaling. CD28 expression was substantially reduced in the proband's T cells, and was diminished to a lesser degree in the T cells of the younger sibling, who has a milder clinical phenotype. Defects in both T and B cell compartments were observed, including absent central memory CD8+ T cells, and decreased frequencies of total and class-switched memory B cells. FOXP3+ regulatory T cells (Treg) were also quantitatively decreased, and furthermore CD25 expression within the Treg subset was substantially reduced. These data confirm the pathogenicity of this novel loss-of-function (LOF) variant in *CARMIL2* and expand the genotypic and phenotypic spectrum of CIDs associated with EBV-SMTs.

## Introduction

CARMIL2 (capping protein regulator and myosin 1 linker (2) deficiency is a rare autosomal recessive combined immunodeficiency (CID) characterized by impaired T cell activation, differentiation, and migration. CARMIL2 (RLTPR) is essential for CD28-mediated T cell co-stimulation, and not only promotes actin polymerization at the immunological synapse and leading edge of migrating cells, but can also modulate signaling in B, NK, and some myeloid cells. The clinical phenotype is heterogeneous and includes recurrent infections, eczematous or psoriaform dermatitis, inflammatory bowel disease (IBD), and malignancy (1–9). We report a novel homozygous *CARMIL2* LOF variant presenting with growth failure and Epstein Barr Virus smooth muscle tumors (EBV-SMTs) in two Saudi Arabian brothers born to consanguineous parents. We provide functional and immunophenotypic evidence establishing pathogenicity. To the best of our knowledge, this is the third reported *CARMIL2* variant associated with EBV-SMTs (7).

## Methods

Trio whole-exome sequencing (WES) was performed on the proband and his parents by Baylor Genetics (Houston, TX), using RefSeq NM_001843.3. T cell proliferation to stimulation with soluble anti-CD3 and soluble anti-CD3 with anti-CD28 was performed clinically using an Edu®-based flow cytometric assay (10). CARMIL2 and DOCK8 protein expression were analyzed by intracellular flow cytometry, using anti-RLTPR clone EM-53 (Invitrogen, Carlsbad, CA) and PerFix-NC® kits [Beckman Coulter [BC], Brea, CA], and a polyclonal C-terminal-specific anti-DOCK8 antibody (Abcam, Cambridge, MA) revealed by a secondary antibody (mouse anti-rabbit IgG AF®647, Jackson ImmunoResearch, West Grove, PA). The DOCK8 flow assay used the KIT True-Nuclear^TM^ transcription factor buffer set (BioLegend, San Diego, CA). Regulatory T cells (Treg) were analyzed using the Human Treg Whole Blood Staining Kit (Invitrogen) with anti-FOXP3 clone PCH101 (Invitrogen) and anti-CD25 clone BC96 (BioLegend, San Diego, CA) antibodies. Detailed T cell and B cell immunophenotyping was performed using multiparametric flow cytometric assays (Supplementary Table 1). A minimum of 5000 CD3+ or CD3- lymphocytes were collected for analysis of T and B cell subsets. Samples were acquired on a Cytoflex cytometer (BC, Brea, CA) and analyzed with Kaluza C-v1.1 (BC). The immunophenotyping has been validated as part of a clinical diagnostic panel and there were challenges in obtaining repeated blood draws; therefore, multiple replicates of the patient sample for these assays were not performed.

## Results and Discussion

P1 is a 12-year-old Saudi Arabian male, born to consanguineous parents, with Crohn's disease (presenting with chronic diarrhea and weight loss at 3 years, but diagnosed at 5 years), growth hormone (GH) deficiency, eczema, asthma, and severe growth failure refractory to exogenous GH and IBD therapy (on presentation 11 kg/Z −10, 111 cm/Z −5). He contracted pneumonia requiring hospitalization at 8 years of age. During treatment with azathioprine and inhaled corticosteroids, he developed chronic thrush. At 11 years, he presented with right eye ptosis caused by multifocal EBV-SMTs involving the brain, and was discovered to have simultaneous tumors in the L5-S1 para-spinal region, lungs, colon, gallbladder, and kidneys (Supplementary Figure 1). Resection of the frontal lobe mass demonstrated sheets of moderately-pleomorphic spindle cells positive for actin, caldesmon, and EBV-encoded small RNA, confirming the diagnosis of EBV-SMTs (Supplementary Figure 2). Human immunodeficiency virus PCR was negative. He had low-level CMV and EBV DNAemia, which improved after discontinuing immunosuppressants.

He was initially treated with rapamycin (Sirolimus®) as mTOR activation has been implicated in EBV-SMT tumorigenesis (11). Five days later, he presented with sepsis secondary to disseminated *Mycobacterium chelonae*, which was controlled with antibiotics (amikacin, clarithromycin, and linezolid). Sirolimus was discontinued, and he was transferred to our facility. For pain and local control of unresectable disease, he received stereotactic ablative radiotherapy to specific intracranial, thoracic, and pelvic tumors, which initially stabilized. His course was complicated by pneumatosis intestinalis, which resolved with conservative management, and progression of the cerebellar and skull base lesions with subclinical seizures and hyponatremia requiring steroids, hypertonic saline, and anti-epileptic medications. He also developed Candidemia, *Cryptosporidium* enterocolitis, and moderate peripheral eosinophilia (likely reactive to mycobacterial infection, but possibly potentiated by actin dysregulation leading to Th2-skewing) (12).

After he was stabilized, P1 received a TCRαβ+/CD19+ depleted peripheral blood stem cell (PBSC) transplantation from a matched unrelated donor (MUD). A decision was made to lympho-deplete the graft to mitigate the risk of graft-versus-host disease (GvHD), given only PBSCs were available, and a reduced toxicity preparative regimen was required in light of his pre-transplant functional status. Conditioning consisted of busulfan (target AUC 1900–2400 μmol^*^min/day), fludarabine, thiotepa, rATG, and rituximab. Post-HCT course was complicated by engraftment syndrome and respiratory failure attributed to *Pneumocystis jirovecii* (PJP) pneumonia, CMV pneumonitis, progression of pulmonary tumors, and possible idiopathic pulmonary syndrome, resulting in death at D+32. Immune monitoring at D+30 (while he was on systemic corticosteroids) revealed significant lymphopenia (CD45+ALC 31 cells/uL), complete donor chimerism in the myeloid lineage, and only 46% donor chimerism in peripheral blood CD3+ T cells.

**Figure 1:**
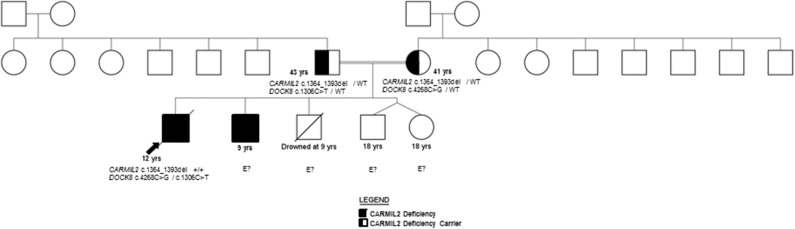
Pedigree.

P2 is the 9-year-old brother of P1 (Figure 1), with history of transient diarrhea (but reportedly normal GI endoscopies), asthma, and GH deficiency (16kg/Z −5, 106 cm/Z −5). At 8 years of age, he developed chronic headaches, and MRI showed findings consistent with a right occipital meningioma. He transferred care to the U.S. a few months after the diagnosis of the proband, at which time a CT scan demonstrated multiple lesions in the lungs, liver, and spleen. A liver biopsy in this patient also revealed the presence of EBV-SMTs, similar to the older sibling. As such, P2 will also require allogeneic HCT as soon as possible. His improved performance status at present may increase the likelihood of a favorable response; however, P1's outcome suggests urgency is required.

Both siblings' immunizations were current on presentation to our facility, and they received the neonatal BCG vaccine without clinical complications. Family history includes two healthy dizygotic twin siblings and an older sibling who died from an accidental cause. The father has insulin-dependent diabetes mellitus diagnosed at 13 years, and the mother is healthy (Figure 1). A maternal aunt was diagnosed with adult-onset thyroiditis.

The family history of consanguinity, multiple EBV-SMTs, and immune dysregulation were strongly concerning for a primary immunodeficiency/ immune dysregulatory disorder, and genetic analysis was pursued. In the proband, a SNP microarray detected 22 regions of homozygosity encompassing 273,807,963 base pairs and 223 recessive genes, two of which were associated with immunodeficiency (*CARMIL2* and *DOCK8*). WES identified a novel homozygous *CARMIL2* c.1364_1393del (p.Gln455_Leu464del; NM_0010138381) variant and compound heterozygous variants of uncertain significance (VUS) in *DOCK8* (c.4268C>G, p.Ala1423Gly and c.1306C>T, p.Arg436Trp; NM_203447.3). The p.Ala1423Gly *DOCK8* variant has a minor allele frequency (MAF) of 0.0008% in gnomAD and is predicted to be disease-causing by MutationTaster® and possibly damaging by PolyPhen-2. The p.Arg436Trp *DOCK8* variant has a MAF of 0.0028% and is predicted to be deleterious by SIFT, disease-causing by MutationTaster®, and probably damaging by PolyPhen-2. Each parent is a carrier of the *CARMIL2* variant and one *DOCK8* variant (Figure 1). *CARMIL2* p.Gln455_Leu464del results in the loss of ten amino acids from the leucine-rich repeat (LRR) domain, which is highly-conserved between species, maintains protein stability, and mediates protein-protein interactions (1, 13), thus favoring the pathogenicity of this variant. There are a variety of *in silico* prediction tools used for assessment of the impact of non-synonymous amino acid changes or sequence alterations on protein function and expression. The variants in the proband were evaluated with SIFT®, MutationTaster® and PolyPhen-2®, all of which use different algorithms and therefore, have different classifications for pathogenicity or benign status of a variant (14–18). While an analysis of the pros and cons of each of these prediction tools is beyond the scope of this report, the additional immunological analyses performed herein along with the clinical phenotype provided more objective and robust verification of the pathogenic classification of this variant, which was previously classified as a variant of uncertain significance (VUS).

Missense variants in the LRR have been previously demonstrated to cause clinical and biochemical phenotypes. *CARMIL2* LOF was demonstrated in three kindred harboring homozygous *CARMIL2* variants Leu638His (1), Leu372Arg (9), and Leu525Glu (9), respectively, who presented with typical infectious sequelae and dermatitis during childhood (1, 9). Functional interrogation of mice homozygous for the *CARMIL2* variant associated with p.Leu432Pro suggested defective coupling of CD28 to PKCθ (protein kinase C theta) and CARMA1 (CARD11) (19). Genetic testing (WES) was ordered on P2, however, the results were unavailable after a period of 3 months. The diagnosis of CARMIL2 deficiency in the brother (P2) was established by protein analysis as discussed below.

Given the novel *CARMIL2* variant and associated phenotype, we performed additional immunologic investigations including functional assessment of the T cell compartment, evaluation of CARMIL2 and DOCK8 protein expression by flow cytometry, and detailed T cell, regulatory T cell (Treg), and B cell immunophenotyping.

**Figure 2:**
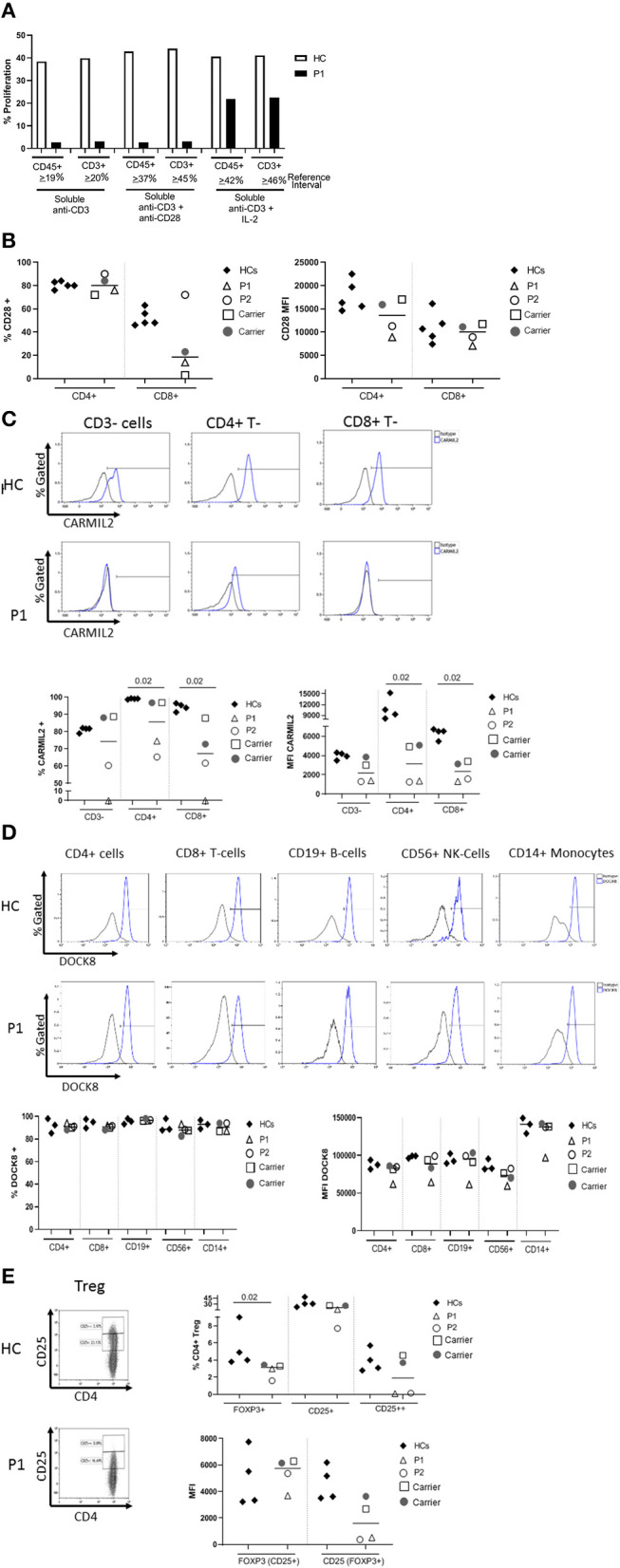
**(A)** Flow cytometric evaluation of T lymphocyte proliferation. T cell proliferation after stimulation with soluble anti-CD3, soluble anti-CD3 and anti-CD28, and soluble anti-CD3 with recombinant interleukin-2 (IL-2) was measured using an Edu-based® flow assay with CD45 and CD3 as markers for total lymphocytes and CD3+ T cells, respectively. Data is expressed as either % CD45+ lymphocytes relative to total CD45+ lymphocytes, and % CD3+ T cells relative to total CD3+ T cells. Data is shown with an experimental healthy control. This was performed as a clinical test validated in 101 healthy controls, and therefore multiple replicates were deemed unnecessary as clinical assays are stringently analytically validated for regulatory oversight. Further, the volume of blood required to repeat this clinical testing was contraindicated in the patient. **(B)** CD28 expression on CD4+ and CD8+T cells by flow cytometry. **(C)** CARMIL2 protein expression in CD4+ and CD8+T cells by flow cytometry. **(D)** DOCK8 protein expression in peripheral blood mononuclear cell subsets by flow cytometry. **(E)** Regulatory T-cell (Treg) phenotyping. Flow cytometric assays shown in **(B–E)** were performed on the proband (P1), his younger affected sibling (P2), their parents (carriers), and HCs. Assays were performed at least twice on each sample yielding consistent results. Samples from P1 and P2 were inadequate for further replicates. The frequency of CD28+ T cells is shown in the first graph, and the median fluorescence intensity (MFI) is shown in the second graph (right). The parents also show decreased frequency of CD28+ CD8+ T cells, though the MFI shows overlap between the two patients and a couple of healthy controls. CARMIL2 protein expression was assessed in both CD4+ and CD8+ T cells, as well as CD3-negative lymphocytes (B and NK cells). For DOCK8 protein expression, while the frequency of DOCK8 is comparable between P1 and P2, parents (carriers), and healthy controls, the MFI is decreased in all lymphocyte subsets and monocytes in P1. The expression (MFI) of CD25 in FOXP3+ Tregs is substantially reduced in both P1 and P2.

The proband's T cell proliferation to stimulation with soluble anti-CD3, soluble anti-CD3 with anti-CD28, and soluble anti-CD3 with exogenous IL-2 (Figure 2A) was significantly blunted compared to healthy controls (HC), especially for soluble anti-CD3 and anti-CD3+anti-CD28. In P1 and the experimental healthy control, there is no difference between the response to soluble anti-CD3 alone or soluble anti-CD3+anti-CD28, which is in contrast to the reference interval derived from over 100 healthy controls. IL-2 partially rescued T cell proliferation, consistent with prior studies (7). CD28 protein expression (median fluorescence intensity, MFI) on P1 and P2's CD4+ T cells was reduced compared to HCs and parents, but the expression of CD28 on CD8+ T cells was normal in both P1 and P2 and parents, comparable to the HCs (Figure 2B). In the CD4+T cell subset, though P2 showed a slightly higher frequency of CD28+ cells than P1, the data for both patients and parents was comparable to the HCs. On the other hand, the frequency of CD28+CD8+ T cells was decreased in P1 and both parents, but normal in P2 (Figure 2B).

CARMIL2 protein expression in CD4+ and CD8+T cells is shown in Figure 2C. In P1, the frequency of CARMIL2+ CD4+T cells was decreased, but not absent; however, CARMIL2 expression (MFI) in these cells was substantially diminished. More importantly, there were no CD8+T cells or CD3-negative lymphocytes (B and NK cells) expressing CARMIL2 (Figure 2C). These findings correlate well with reduced T cell proliferation to soluble anti-CD3 and anti-CD28, and the clinical phenotype of EBV-SMTs.

P2, who has a less aggressive clinical phenotype particularly with regard to infections, had comparable CARMIL2 expression to P1 in C3-negative lymphocytes, CD4+ T cells, and CD8+T cells. However, the frequency of CD4+ and CD8+T cells expressing CARMIL2 was markedly reduced compared to HCs. Interestingly, the parents as heterozygous carriers of the *CARMIL2* variant showed lower expression of CARMIL2 in CD4+ and CD8+ T cells compared to HCs, but not in CD3-negative lymphocytes. (Figure 2C), but they had no relevant clinical phenotype. There were no significant differences in CD28 (Figure 2B) and CARMIL2 expression (MFI) (Figure 2C) observed between the proband and his younger sibling despite the differences in clinical phenotype, which may reflect age-related progression of disease, and potentially other epigenetic factors.

DOCK8 protein expression was also analyzed (Figure 2D) in P1, as he had compound heterozygous variants of uncertain significance. DOCK8 modulates actin polymerization and cytoskeletal remodeling in leukocytes (20, 21), and consequently LOF due to the combination of variants could potentially contribute to the clinical phenotype. P1 demonstrated reduced expression of DOCK8 within monocytes, T, B, and NK cells compared to his kindred (P2 and parents) and HCs, although the frequency of DOCK8-expressing cells appeared normal (Figure 2D). While he lacks many of the clinical and immunologic features of complete DOCK8 deficiency (20, 21), partial LOF resulting from the compound heterozygous variants could possibly modulate the clinical phenotype (perhaps contributing to the enhanced severity of atopy compared to his brother).

T cell subset analysis (Table 1) revealed increased naïve CD4+CD45RA+T cells for age and a complete absence of CD8+ central memory T cells (Tcm) in P1 and P2, which has been previously reported in CARMIL2 deficiency (9). P1 also demonstrated relative expansion of CD8+ effector memory T cells (Tem) and T-effector cells re-expressing CD45RA+ (TEMRA). Most of P1's CD8+T cells expressed CD57 (71%, compared to 17% in HCs), a marker of terminal differentiation and senescence. Collectively, these findings likely represent an ineffective immune response to chronic mycobacterial infection and EBV-SMTs.

**Table 1:** T/B/NK quantitation and detailed T cell subset phenotyping.

	**P1**	**P2**	**Control**
**Lymphocyte subset quantitation**			Pediatric reference interval 95% CI^*^
CD45+/CD14- lymphocytes (cells/μL)	6,741	3,746	1,561–4,630
CD3+ T cells (% lymphocytes, cells/μL)	85%, 5,774	70%, 2,630	58–78%, 1,204–2,889
CD3+CD4+ T cells (% lymphocytes, cells/μL)	40%, 2,695	43%, 1,597	32–46%, 505–1,644
CD3+CD8+ T cells (% lymphocytes, cells/μL)	40%, 2,715	19%, 717	18–37%, 336–1,296
CD19+/CD20+ B cells (% lymphocytes, cells/μL)	12%, 797	27%, 1,276	13–29%, 215–1,230
CD16++/CD56+ NK cells (% lymphocytes, cells/μL)	0.8%, 57	ND	4–22%, 102–827
**T-Cell subset phenotyping**			Median of 38 HCs^**^
CD4+CD45RA+ (%CD4+)	83	90	38
CD8+CD45RA+ (%CD8+)	77	76	52
Naïve^†^ CD4+ (%CD4+)	36	42	48
Naive CD8+ (%CD8+)	8	22	18
TEMRA CD4+ (%CD4+)	0.1	0.4	0.7
TEMRA CD8+ (%CD8+)	17	2.6	5
CD4+CD45RO+ (%CD4+)	8	3	43
CD8+CD45RO+ (%CD8+)	5	3	18
Central Memory^§^ CD4+ (%CD4+)	11	10	26
Central Memory CD8+ (%CD8+)	0	0	3
Effector Memory^¶^ CD4+ (%CD4+)	14	27	15
Effector Memory CD8+ (%CD8+)	28	20	21
**Regulatory T-Cell quantitation**			**median of 4 HCs**
CD4+FOXP3+ (%CD4+)	3	2	9
CD4+CD25+ (%CD4+)	17	8	23
CD4+CD25++ (%CD4+)	0.1	0.2	4

* *CI, confidence interval*.

** *HC: A pediatric reference interval for these subsets is in the process of being established, and therefore, comparison was made to healthy adults ages 19–41 + years*.

† *Naïve T cells CD45RA+CD62L+CCR7+*.

*TEMRA, T-effector memory cells re-expressing CD45RA+: CD45RA+CD62L-CCR7-*.

§ *Central Memory T cells: CD45RO+CD62L+CCR7+*.

¶ *Effector Memory T cells: CD45RO+CD62L-CCR7-*.

*ND, not done*.

The frequency of FOXP3+Tregs was decreased (Figure 2E, Table 1), similar to previous reports described in other CARMIL2-deficient patients, and supported by the critical role of CD28 in Treg lymphopoiesis (1, 9, 22, 23). Furthermore, expression of CD25 on FOXP3+Tregs was substantially diminished (Figure 2E), which likely compromises Treg suppressor function and contributes to P1's immune dysregulatory phenotype with IBD.

B cell subset analysis (Table 2) revealed decreased total memory B cells and plasmablasts (CD19+CD27+) with significantly reduced class-switched memory B cells, which may be due to aberrant T cell help through impaired CD28-mediated co-stimulation, as well as intrinsic B cell defects in NFκB activation upon BCR stimulation (9). The younger sibling (P2) has some preserved switched memory B cells compared to the proband (P1), which may represent an age-associated loss over time, especially as the proband had a more aggressive clinical phenotype when he presented. The role of the proband's reduced DOCK8 expression in impairing memory B cell formation also cannot be excluded (21).

**Table 2:** B-Cell subset phenotyping.

**B-cell subset**	**P1**	**P2**	**Median of 38 HCs**
Naive B cells, CD19+CD27–IgM+IgD+ (%CD19+)	85	86	71
Total memory B cells & plasmablasts, CD19+CD27+ (%CD19+)	2	9	31
IgM memory B cells, CD19+CD27+IgM+IgD– (%CD19+)	0.5	0.9	2
Switched memory B cells, CD19+CD27+IgM–IgD– (%CD19+)	0.5	3	10

Quantitative immunoglobulins were obtained in the proband and showed an elevated serum IgM (240 mg/dl) with undetectable IgE, likely reflecting the isotype-switch defect. Alternate pathways of class switching in the mucosa and other secondary lymphoid tissue may explain the apparently paradoxical elevation in IgA (665 mg/dl) (24–26). Quantitatively normal IgG (1130 mg/dl) may reflect the presence of long-lived plasma cells in the bone marrow, which developed before the evolving class-switch defect.

These data conclusively support the pathogenicity of this novel *CARMIL2* variant, p.Gln455_Leu464del, affecting both the numbers and function of T cell and B cell subsets. While we were unable to evaluate the healthy older siblings since they resided in a different country, the genetic, immunologic, and phenotypic data available for the affected siblings and their parents is consistent with an autosomal recessive, completely-penetrant LOF in *CARMIL2*, similar to previous familial segregation analyses (1–9). Shared phenotypic findings in the affected brothers include severe growth failure refractory to exogenous growth hormone and IBD therapy (in the case of the proband), and EBV-SMTs.

There is a paucity of information on long-term prognosis given the relatively recent description of this disorder in 2016, and the predilection for EBV-SMTs rather than EBV lymphoproliferative disease remains unclear (27, 28). The complete absence of central memory CD8+T cells (Tcm) in both of these patients is striking. CD8+ Tcm lymphopenia and susceptibility to EBV SMTs have also been observed in GATA2 deficiency (29), and the contribution of this immunologic finding to tumorigenesis warrants further investigation. There is some evidence to suggest that the presence of EBV-SMTs is associated with a worse prognosis, with three of four patients succumbing to chemotherapy-refractory disease and one showing disease progression at the time of report (7). Surgical or stereotactic radiation-induced reductions in tumor burden can aid in preparation for more definitive therapy, but are in themselves insufficient for disease control (11). It is logical to surmise that the only effective management, especially in the context of EBV-SMTs, is through robust immune reconstitution by hematopoietic cell transplantation (HCT). One patient with GATA2 deficiency and EBV-SMTs showed stability or involution of the tumors with complete loss of positron emission tomography (PET) enhancement 3 years after allogeneic-HCT (30). More recently, a patient with CARMIL2 deficiency and EBV-SMT has been transplanted (Dr. F. Hauck, Munich, Germany, personal communication) and long-term data on this patient is awaited.

The challenges of attempting allo-HCT in patients with significant comorbidities associated with CARMIL2 deficiency emphasizes the need for early diagnosis, especially in susceptible individuals, e.g., where the population frequency of consanguinity is high. The presence of early-onset immune dysregulation should instigate a thorough work-up for an underlying inborn error of immunity, including genetic analysis. These cases also underscore the need for systematic evaluation of PIDD patients with EBV-SMTs to identify the functional immunologic defects which promote their development and to guide therapy.

## Ethics Statement

We have written, informed consent for the publication of this case report using an IRB-approved document obtained via translation into the native language of the family by the legal guardian of the patient since the patient is below age of legal consent. We also verbalized assent by the patient in accordance with our IRB protocol.

## Author Contributions

JY, AG, CR, SM, BP, HR, MA, EV, TC, and RA all contributed meaningfully to authorship of the manuscript. CR performed the CARMIL2, DOCK8, and Treg flow cytometry experiments in the Diagnostic Immunology Laboratory of Nationwide Children's Hospital. SM reviewed the pathology sections and provided interpretation on the histopathology.

## Conflict of Interest

The authors declare that the research was conducted in the absence of any commercial or financial relationships that could be construed as a potential conflict of interest.

## Supplementary Material

Supplementary Figure 1 Timeline depicting the proband's clinical course.

Supplementary Figure 2 Light microscopic findings of EBV-SMTs. **(A)** Low power magnification (x100) from the hematoxylin and eosin (H&E) sections of the tumor from patient 1 (P1) showing higher cellularity to right and less cellular area on left. **(B)** High power magnification (x600) from H&E section of the more cellular area demonstrating closely packed round and oval cells, and a mitotic figure (arrow). **(C)** Medium power magnification (x200) of H&E section of the biopsy from patient 2 (P2) demonstrating a moderately cellular spindle cell tumor similar to that seen in areas of tumor from P1. **(D)** High power (x600) of H&E section from P2 tumor demonstrating spindle cells, some with blunted nuclei. **(E)** There is diffuse cytoplasmic staining of neoplastic cells with the antibody to smooth muscle actin (x200 magnification) in the tumor from P2. **(F)** *In-situ* hybridization to EBER of tumor from P2 demonstrates diffuse nuclear positive staining of neoplastic cells (x200 magnification). Inset shows high power magnification (x600) of the positive nuclei of neoplastic cells. The histopathology of the EBV-SMTs was not significantly different between the two patients.

Supplementary Table 1 Antibodies used for multiplex flow cytometry assays.
