# Supplementary figures and images for: A Novel Pathogenic Variant in CARMIL2 (RLTPR) Causing CARMIL2 Deficiency and EBV-Associated Smooth Muscle Tumors
Source: Front Immunol. 2020 Jun 18;11:884. doi: 10.3389/fimmu.2020.00884 (PMC7314954; doi:10.3389/fimmu.2020.00884)

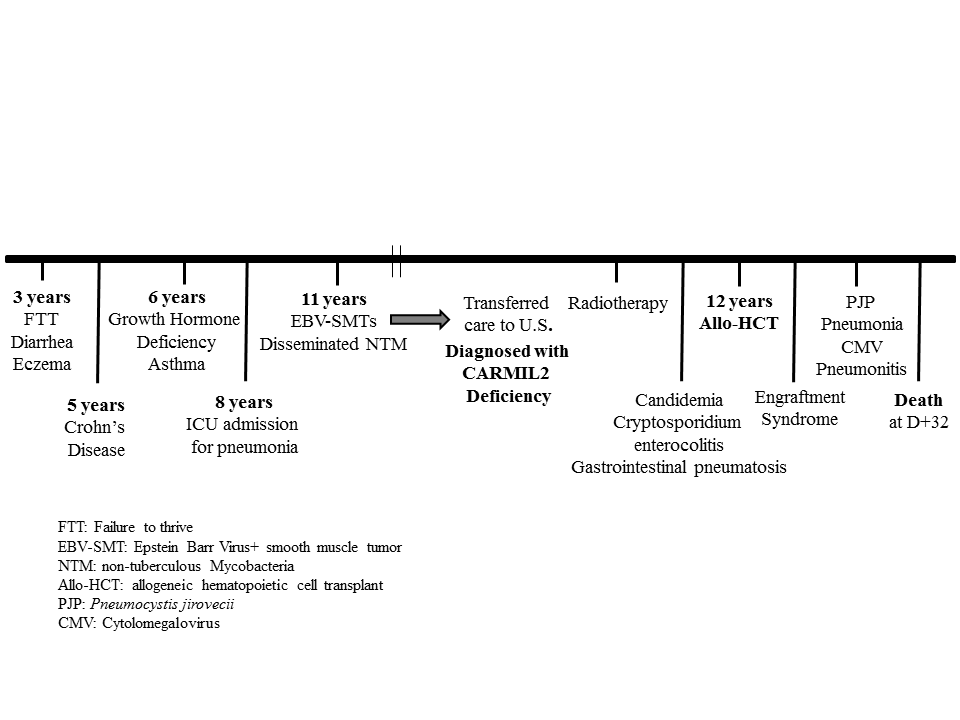

Supplement: Supplementary Figure 1 — Timeline depicting the proband's clinical course. [file Image_1.tif]

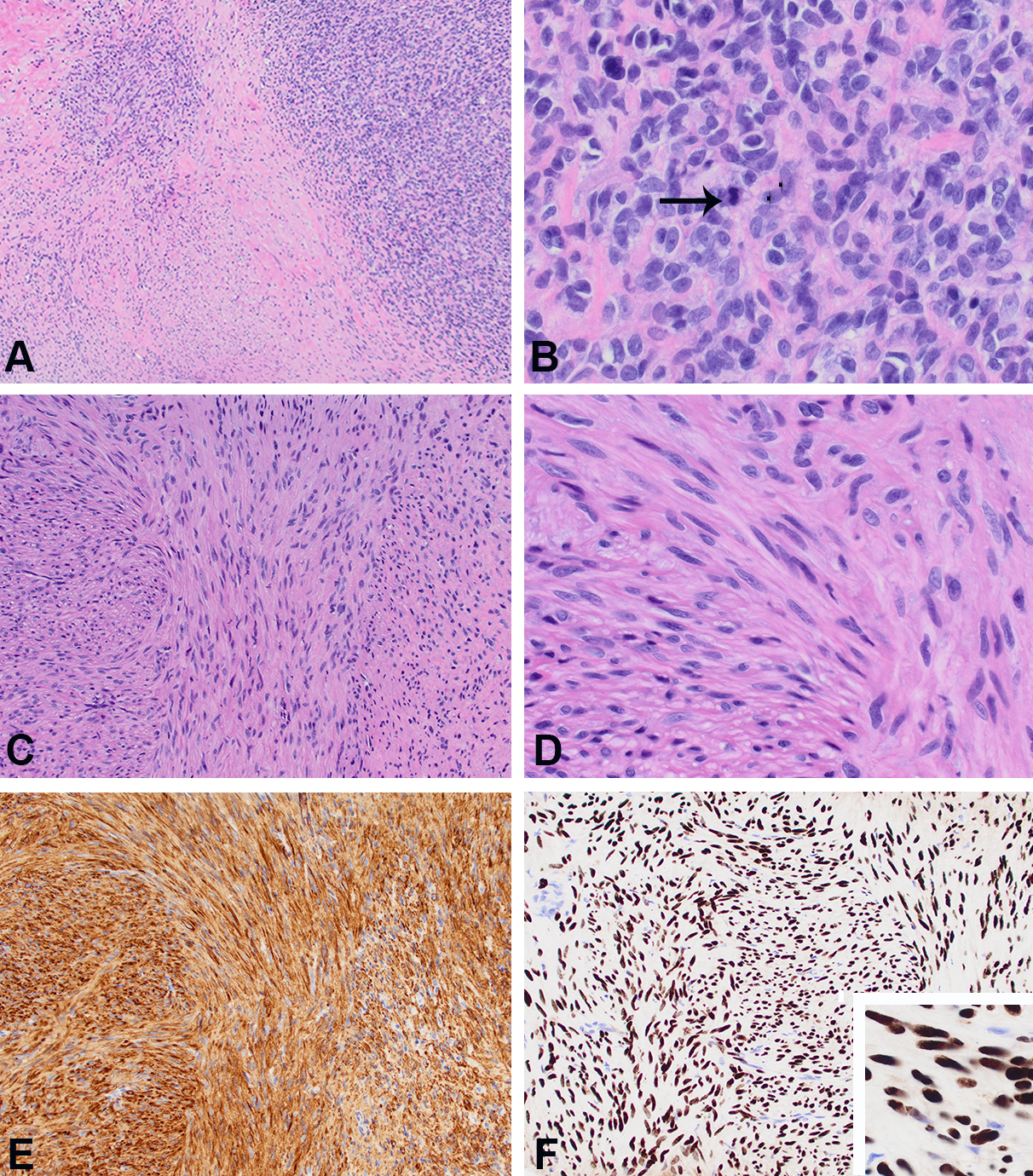

Supplement: Supplementary Figure 2 — Light microscopic findings of EBV-SMTs. (A) Low power magnification (x100) from the hematoxylin and eosin (H&E) sections of the tumor from patient 1 (P1) showing higher cellularity to right and less cellular area on left. (B) High power magnification (x600) from H&E section of the more cellular area demonstrating closely packed round and oval cells, and a mitotic figure (arrow). (C) Medium power magnification (x200) of H&E section of the biopsy from patient 2 (P2) demonstrating a moderately cellular spindle cell tumor similar to that seen in areas of tumor from P1. (D) High power (x600) of H&E section from P2 tumor demonstrating spindle cells, some with blunted nuclei. (E) There is diffuse cytoplasmic staining of neoplastic cells with the antibody to smooth muscle actin (x200 magnification) in the tumor from P2. (F) In-situ hybridization to EBER of tumor from P2 demonstrates diffuse nuclear positive staining of neoplastic cells (x200 magnification). Inset shows high power magnification (x600) of the positive nuclei of neoplastic cells. The histopathology of the EBV-SMTs was not significantly different between the two patients. [file Image_2.tif]
