# Supplementary material for: A Novel Pathogenic Variant in CARMIL2 (RLTPR) Causing CARMIL2 Deficiency and EBV-Associated Smooth Muscle Tumors
Source: Front Immunol. 2020 Jun 18;11:884. doi: 10.3389/fimmu.2020.00884 (PMC7314954; doi:10.3389/fimmu.2020.00884)
Supplement: Supplementary Table 1 — Antibodies used for multiplex flow cytometry assays. [file Table_1.docx]

| **Specificity** | **Fluorochrome- Manufacturer** | **Clone** |
| --- | --- | --- |
| CD14 | APC-AF700- Beckman | RM052 |
| CD3 | FITC- Sysmex | UCHT1 |
| CD4 | APC-Cy7- Sysmex | MEM-241 |
| CD8 | Pacific Blue- Sysmex | MEM-31 |
| CCR7 | PE- Beckman | G043H7 |
| CD62L | ECD- Beckman | DREG56 |
| CD27 | BUV395- BD | L128 |
| CD45RO | PC7- Beckman | UCHL1 |
| CD45RA | APC-Beckman | 2H4 |
| CD45 | Pacific Orange- Sysmex | 2D1 |
| CD57 | PE- BD | NK-1 |
| CD28 | BUV395- BD | L293 |
| TCR α/β | PE- Beckman | IP26A |
| TCR γ/δ | PC7- Beckman | IMMU510 |
| CD3 | PC5.5- Beckman | UCHT1 |
| CD19 | PE-Cy7- Sysmex | LT19 |
| IgA | FITC- Miltenyi | IS11-8E10 |
| IgG | PE- BD | G18-145 |
| IgD | APC- Beckman | IA6-2 |
| IgM | Pacific Blue- Beckman | SA-DA4 |
| CD27 | BUV395- BD | L128 |
| CD21 | PE- Beckman | BL13 |
| CD24 | ECD- Beckman | ALB9 |
| CD10 | APC- Beckman | MEM-78 |
| CD38 | APC-AF700- Beckman | LS198 |
| CD20 | APC- Sysmex | 2H7 |

**Supplementary Table 1.** Antibodies used for multiplex flow cytometry assays
